# Can an Integrated Approach Reduce Child Vulnerability to Anaemia? Evidence from Three African Countries

**DOI:** 10.1371/journal.pone.0090108

**Published:** 2014-03-05

**Authors:** Kendra Siekmans, Olivier Receveur, Slim Haddad

**Affiliations:** 1 HealthBridge, Ottawa, Ontario, Canada; 2 Department of Nutrition, Université de Montréal, Montréal, Québec, Canada; 3 University of Montreal Hospital Research Centre (CRCHUM), Université de Montréal, Montréal, Québec, Canada; Institut Pasteur, France

## Abstract

Addressing the complex, multi-factorial causes of childhood anaemia is best done through integrated packages of interventions. We hypothesized that due to reduced child vulnerability, a “buffering” of risk associated with known causes of anaemia would be observed among children living in areas benefiting from a community-based health and nutrition program intervention. Cross-sectional data on the nutrition and health status of children 24–59 mo (N = 2405) were obtained in 2000 and 2004 from program evaluation surveys in Ghana, Malawi and Tanzania. Linear regression models estimated the association between haemoglobin and immediate, underlying and basic causes of child anaemia and variation in this association between years. Lower haemoglobin levels were observed in children assessed in 2000 compared to 2004 (difference -3.30 g/L), children from Tanzania (-9.15 g/L) and Malawi (-2.96 g/L) compared to Ghana, and the youngest (24–35 mo) compared to oldest age group (48–59 mo; -5.43 g/L). Children who were stunted, malaria positive and recently ill also had lower haemoglobin, independent of age, sex and other underlying and basic causes of anaemia. Despite ongoing morbidity, risk of lower haemoglobin decreased for children with malaria and recent illness, suggesting decreased vulnerability to their anaemia-producing effects. Stunting remained an independent and unbuffered risk factor. Reducing chronic undernutrition is required in order to further reduce child vulnerability and ensure maximum impact of anaemia control programs. Buffering the impact of child morbidity on haemoglobin levels, including malaria, may be achieved in certain settings.

## Introduction

Childhood anaemia remains a problem of global importance, affecting 273 million children worldwide [1]. The most recent analysis shows that children under five years of age in sub-Saharan Africa have the lowest mean concentrations of haemoglobin and the highest anaemia prevalence [1]. The slow pace of progress in anaemia control efforts is in large part due to its complex aetiology and the inherent challenge of delivering multiple, integrated health and nutrition interventions at scale [2]. Yet the consequences of anaemia for child development and survival are sobering and provide the impetus to find more effective approaches.

**Figure 1:**
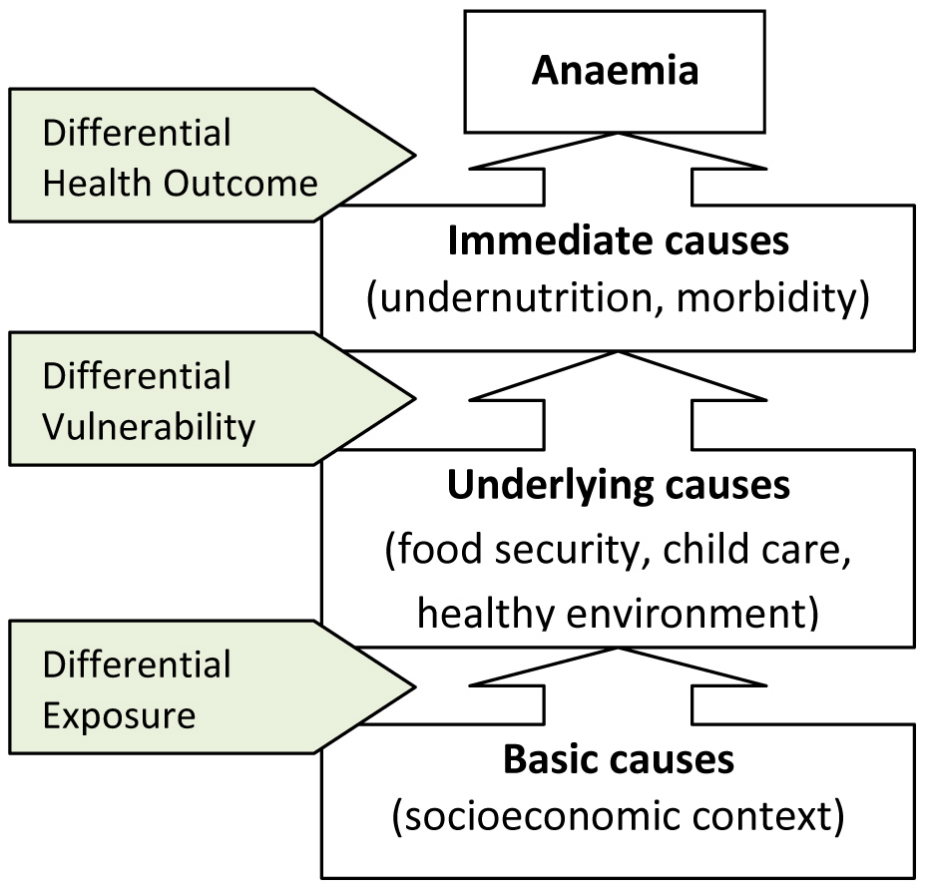
Conceptual framework for the analysis of anaemia risk [Adapted from 3,4].

Traditionally, anaemia control programs have focused on the reduction in prevalence of risk factors as a means of reducing anaemia. Reductions in children's exposure to iron-poor diets and parasitic infections (e.g. malaria or hookworm) are expected to result in reduced anaemia. Similar to the UNICEF theoretical framework for the causes of undernutrition [3], a combination of immediate, underlying and basic causes interact to cause anaemia, as shown in Figure 1. However, many other factors mediate the effect of an exposure to a risk factor on the individual exposed. Children exposed to similar risk factors have different levels of capacity to respond to that exposure, influenced to a large degree by individual, household and community factors that work together in complex ways to either increase or reduce their vulnerability [4]. Therefore our theoretical model also represents the role of differential exposure and differential vulnerability in contributing to differential health outcomes.

The concept of vulnerability encompasses both the external exposure to risk factors and the internal means for coping with those risks without damaging loss [5]. In low and middle-income country contexts, early childhood is a period of high exposure to external stressors, including micronutrient-deficient diets and infectious diseases. Young children are often considered vulnerable since they are more likely to be harmed by these stressors than others in the general population [6]. However, a wide range of factors contribute to the variation observed in the impact of disease on young children, including individual factors (e.g. biological immunity), household factors (e.g. caregiver knowledge) and institutional factors (e.g. adequacy of health services) [7], [8].

Given that exposure, whether to risk factors such as infectious agents or to protective events such as deworming campaigns, interacts with underlying capacities to shape child vulnerability at any particular point in time [6], it is possible to hypothesize the expected effect of an intervention or multiple interventions over time. Even without reducing the level of child exposure to immediate causes of anaemia such as iron deficient diets or malaria infection, anaemia control programs may be able to alter the risk relationship between exposure and effect by enhancing a child's capacity to respond. Capacity is enhanced by community-based health and nutrition interventions that address the underlying and basic causes of anaemia at the household, community and/or institutional levels, thus reducing vulnerability. For example, in a study in Ethiopia, the risk relationship between malaria and mortality changed in intervention communities as a result of teaching mothers with low education levels how to take care of their sick children and supplying them with appropriate drugs for treatment at home [9]. In this case, the intervention reduced the vulnerability of these children to malaria-related mortality by acting at the household capacity level (knowledge of mothers) and the institutional/systemic capacity level (access to malaria medication), without changing the level of exposure of the children to malaria infection.

In contexts where there is delivery of a package of integrated health and nutrition interventions, one would expect a similar buffering (or mitigating) effect on the risk of anaemia associated with undernutrition and morbidity, in addition to a direct reduction in the prevalence of risk factors. By intervening at multiple levels within the causal pathway, public health nutrition programs may enhance the capacity to respond of children, their families and communities, as well as the broader system within which they live. This would reduce child vulnerability to anaemia, regardless of the level of exposure to known risk factors for anaemia.

This study was designed to investigate whether the delivery of multiple, integrated health and nutrition interventions in three African countries acted at multiple levels to enhance capacity and reduce child vulnerability to anaemia. In particular, this study explores how multiple interventions that simultaneously address the immediate, underlying and basic causes of anaemia may modify these risk relationships. Our aim was to determine whether the lower mean Hb associated with established risk factors in 2000 would be similar in 2004. We hypothesized that there would be a “buffering” of risk associated with known causes of anaemia among children living in areas benefiting from a community-based health and nutrition program intervention, due to reduced child vulnerability.

## Methods

### Ethics statement

At the start of the Micronutrient And Health (MICAH) program, ethical consent was obtained from the Ministry of Health in each country for conducting the planned activities, including the baseline and follow-up surveys. Before each survey was conducted, program staff contacted authorities in the area to inform them of the survey purpose and methods. Household members in the selected communities were informed about the purpose and benefits of voluntarily participating in the survey. Verbal consent of the household head or other respondent and the child's mother or primary caregiver was sought at each selected household before the interview and recorded on the questionnaire. Mothers or primary caregivers of each child under five years of age selected to be measured (height, weight, biochemical measures) were informed of the procedures to be taken and if they consented to participate with their child, were asked to bring their child to a central location in their community for measurement. The survey documentation does not state why written consent was not obtained, although we can speculate that this was due to low literacy levels, particularly for women, in the survey communities. The survey protocol was approved by the respective country Ministry of Health ethics committees and no specific concerns raised at that time about the informed consent procedures planned. No financial or material incentives were given to respondents or their children to participate in the survey. Treatment was given to all individuals who were tested and found to be positive for malaria, hookworm, schistosomiasis or anaemia (Hb <110 g/L for children under five years of age).

### Study Context

The data for this study are taken from program evaluations conducted by World Vision in Ghana, Malawi and Tanzania to assess the impact made by the MICAH program. Designed to improve the health and nutritional status of women and children, with a specific focus on vitamin A, iron and iodine deficiencies, the MICAH program delivered an integrated package of health and nutrition interventions to targeted communities. Specific activities included nutrition education, breastfeeding promotion, dietary diversification, micronutrient supplementation and fortification, malaria and other parasitic disease control, water and sanitation promotion, community and health facility level training and local and national level advocacy efforts. Details about interventions delivered in specific countries have been published elsewhere [10] and are available in a supplementary file (see Table S1). Interventions began to be delivered in 1997 in all three countries.

Given the program's integrated approach to health & nutrition interventions delivered at the community level, it was expected to have a direct effect on many of the causes of anaemia in young children. We focused our analysis on anaemia in children 24–59 months of age, since their health and nutrition status is relatively stable and expected to reflect the cumulative effect of their environment during the earlier years of development, including the program's interventions and the various risk factors for anaemia.

Data on the nutrition and health status of children were obtained in 2000 and 2004 from cross-sectional household surveys in program-targeted communities of Ghana, Malawi and Tanzania. Survey methodology was standardized across countries, based on guidelines for multiple indicator cluster surveys [11]. Two-stage cluster sampling with stratified probability sampling methods were used in each country. Data collection included a household interview, biochemical sample collection and clinical examination, including anthropometric measurements.

Household interviews conducted with the child's primary caregiver used a standardized questionnaire that was contextualized for each country. Data on household characteristics included source of drinking water, type of toilet facilities, distance to the nearest health facility and a group of asset variables from which a wealth index was derived using multiple correspondence analysis [12]. Maternal age and education level were also recorded. Data on child characteristics included date of birth based on child health cards (or reported age in months if card not available), sex, and reported illness in the previous two days.

Biochemical samples were collected for a randomly selected sub-sample of children <5 y. Haemoglobin (Hb) concentration was measured using a portable photometer (HemoCue AB, Angelholm, Sweden). Adjusted for altitude [13], a cut-off of <110 g/L was used to define anaemia; moderate-severe anaemia was defined as Hb<100 g/L. Presence or absence of malaria parasites was determined by microscopy of thick blood smears.

Child height was measured to the nearest 0.1 cm using a height board (Malawi 2000 & 2004, Tanzania 2004), microtoise (wall chart, Ghana 2000 & 2004), or portable Harpenden stadiometer (Tanzania 2000). Weight was measured to the nearest 0.1 kilogram using calibrated Salter scales in Ghana and Malawi, and SECA electronic scales in Tanzania. A child's nutritional status was classified as stunted, underweight or wasted when height-for-age (HAZ), weight-for-age (WAZ) and weight-for-height (WHZ) scores were below -2 SD, respectively [14].

### Statistical analysis

We restricted our sample to one child 24–59 mo per household with data on Hb, malaria and anthropometric status. The children in the 2000 sample were expected to have benefited from the MICAH interventions for up to two years whereas the children in the 2004 sample most likely benefited from the interventions from pre-conception onwards. The analysis therefore tests for an association between longer exposure to program interventions in the early years of development and child Hb levels.

To maximize power, data were combined for all countries and years. Multiple linear regression models were built in a series of models using Hb as the dependent variable and blocks of independent variables that were consistent with the conceptual framework. The immediate determinants of child anaemia were stunting (as an indicator of chronic malnutrition and multiple constraints to health), malaria and recent illness. Underlying causes on which the program was expected to have an effect included household water source and toilet type. Basic causes of anaemia included maternal education level, household wealth rank and distance to the nearest health facility. In order to adjust for the variance associated with country and year, these variables were included in all models. Child age group and sex were also included in all models as potential confounders of the association of all risk factors with Hb levels. Very little change in the coefficients was observed as blocks of variables were added; therefore, only the results for the full model are presented.

To test for variation between years in the observed associations, two-way interaction terms were included in the final pooled model for survey year with the three variables considered as immediate causes of anaemia (stunting, malaria, illness) as well as year with country. Interaction terms significant at p<0.15 were considered as evidence for variation between 2000 and 2004 in the association of the risk factor with mean Hb. When little evidence for variation by year was found in the pooled model, country-specific models were run using the full set of variables and including interaction terms for survey year with stunting, malaria and illness. For the two risk factors with evidence of variation by year (malaria and recent illness), marginal means were estimated in each country model to compare mean haemoglobin levels by year and risk factor. Marginal means are the model-estimated means, not the observed means, by level/category of the risk factor. All analyses were carried out using SPSS version 16.0 (SPSS, Inc., Chicago IL).

## Results

Data were available for a total of 1,353 children in 2000 and 1,052 children in 2004. Characteristics of the children are presented in Table 1 by country and year. The distribution by age group was similar for both years in Ghana and Tanzania but in Malawi, there were more children in the youngest age group in 2000 than in 2004. Undernutrition was a problem of public health significance in all three countries. Stunting prevalence was lowest in Ghana but affected over half of children in the Malawi and Tanzania samples in both years. Lower stunting in 2004 compared to 2000 was observed in Ghana and Tanzania. Measures of morbidity were low in prevalence (<13%) and stable over time in Ghana. In Malawi, prevalence of malaria parasitaemia and recent illness was roughly 30% in 2000 but was lower in 2004. Less than 20% of children in Tanzania were malaria positive at both time points and recent illness was lower in 2004 compared to 2000. Access to potable water sources and reported use of private or improved toilets was similar at both time points in all three countries.

**Table 1:** Child and household characteristics by country and survey year (%).

Characteristic		GHANA		MALAWI		TANZANIA		
		2000	2004	2000	2004	2000		2004
		N = 253	N = 296	N = 466	N = 679	N = 634	N = 77	
Age group	24–35 mo	41.5	39.2	50.9	39.8‡	33.6	41.6	
	36–47 mo	32.4	30.7	32.2	35.8	36.3	29.9	
	48–59 mo	26.1	30.1	17.0	24.4	30.1	28.6	
Male		51.0	54.4	50.0	51.7	50.2	51.9	
Stunted (HAZ<-2)		37.2	29.1†	57.7	55.4	65.5	53.2†	
Underweight (WAZ<-2)		19.8	18.6	26.8	7.7‡	20.8	31.2†	
Wasted (WHZ<-2)		8.3	9.5	7.9	0.6‡	1.9	9.1‡	
Malaria positive		11.1	8.1	31.8	13.4‡	10.7	18.2	
Illness in last 2 days		12.3	12.5	29.8	19.0‡	54.1	33.8†	
Protected water source (dry season)		73.9	77.7	85.6	82.9	47.5	42.9	
Private, improved toilet use		38.3	40.5	77.0	79.7†	75.6	63.6‡	
Maternal education, none/non-formal		11.9	14.9	36.1	23.6‡	26.3	23.4	
Health facility >5 km from household^a^		33.2	19.6‡	57.9	25.5‡	9.9	26.0‡	

† p<0.05; ‡ p<0.001 for chi-square test of difference between 2000 and 2004

a Ghana: over 5 km from household; Malawi: more than 2 hr walk (2000) or over 5 km (2004); Tanzania: over 5 km (2000) or more than 2 hr walk (2004).

The prevalence of anaemia in 2000 varied from 59.8% in Ghana to 63.0% in Malawi and 77.7% in Tanzania (see Table 2). The majority of cases were considered moderate in severity (Hb <100 g/L) in all three countries and at both time points. A large decrease in anaemia was observed between 2000 and 2004 in Ghana, but only a small decrease in Malawi and Tanzania. Mean haemoglobin concentration results followed a similar pattern.

**Table 2:** Anaemia severity and mean haemoglobin concentration by country and survey year.

Characteristic		GHANA		MALAWI		TANZANIA	
		2000	2004	2000	2004	2000	2004
		N = 253	N = 296	N = 466	N = 679	N = 634	N = 77
Anaemia severity^a^							
	Mild	24.1	11.8‡	21.5	25.5†	22.6	22.1†
	Moderate	34.0	17.2	38.8	30.2	49.2	41.6
	Severe	1.6	0.7	2.8	2.4	5.7	0
Hb							
	Mean, g/L (SD)	105.0 (15.8)	113.7‡ (13.9)	103.1 (17.6)	105.9† (16.4)	96.9 (17.4)	102.1† (15.0)
	95% CI	103.0, 106.9	112.1, 115.3	101.5, 104.7	104.6, 107.1	95.6, 98.3	98.8, 105.5

a Anaemia severity cutoffs: mild Hb 100–109 g/L, moderate Hb 70–99 g/L, severe Hb <70 g/L

† p<0.05; ‡ p<0.001 for chi-square test (anaemia) or t-test (mean Hb) of difference between 2000 and 2004.

**Figure 2:**
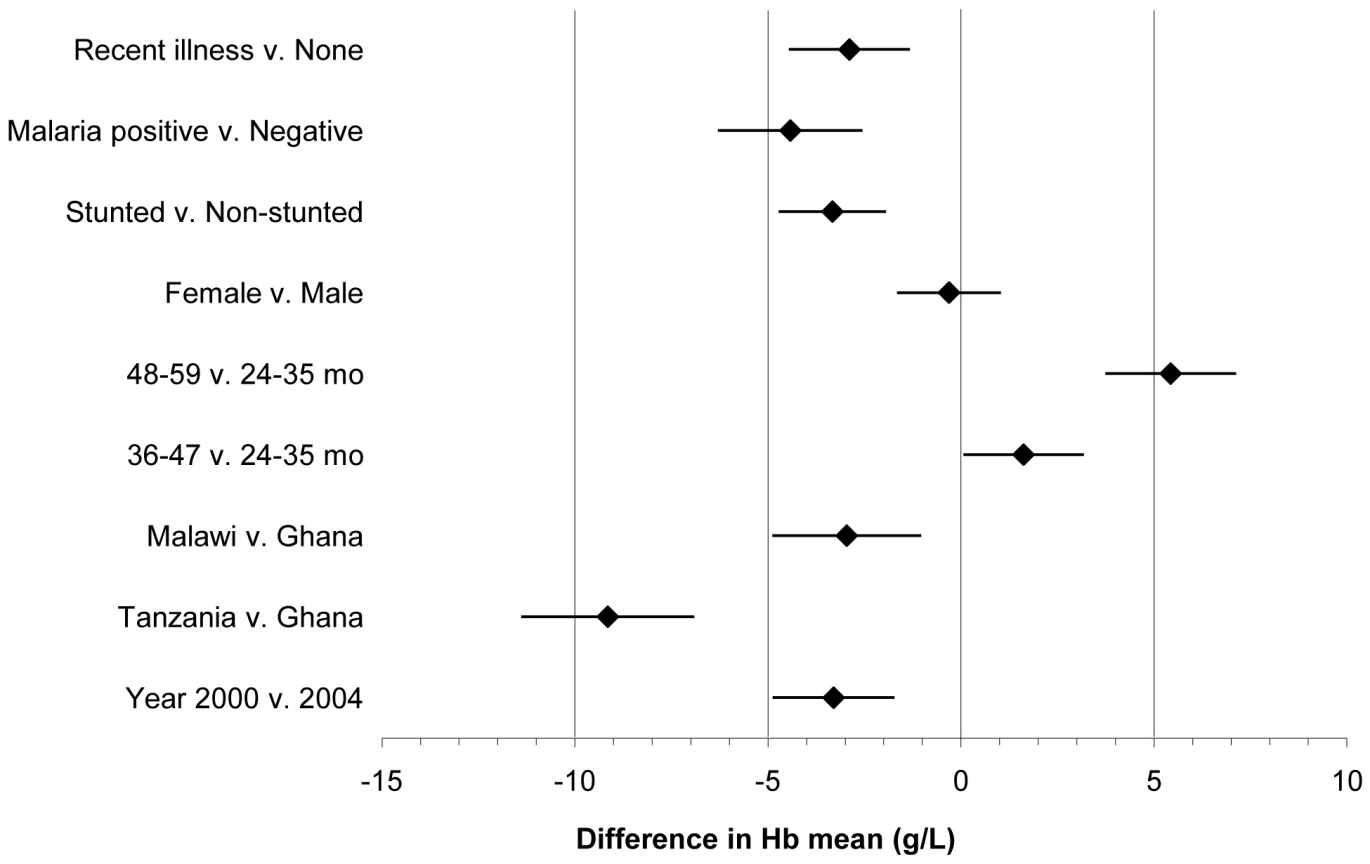
Difference in mean Hb (g/L) with 95% CI based on multiple regression model (N = 2405; model adjusted for potable water source, toilet type, wealth rank, maternal education and distance to health facility).

Estimates of the magnitude of association with mean haemoglobin for known risk factors for anaemia using a pooled data set are presented in Figure 2. Children assessed in 2000 had significantly lower haemoglobin levels than children in 2004, with a difference of -3.30 g/L (95% CI −4.84, −1.76). Children from Tanzania and Malawi also had lower mean haemoglobin levels compared to children in Ghana. Children in the older two age groups had significantly higher haemoglobin levels compared to the youngest group, particularly children 48–59 mo (mean difference 5.43 g/L, 95% CI 3.77, 7.09).

Besides these variations in mean haemoglobin, only the variables that are conceptually considered immediate causes of anaemia had a significant association with mean haemoglobin levels. Estimates of adjusted means indicate significantly lower haemoglobin levels in children who are stunted, malaria positive and recently ill, even when controlling for age, sex and other underlying and basic causes of anaemia. Notably, the difference in mean haemoglobin between children 48–59 mo and children 24–35 mo is of equal or greater magnitude compared to the difference associated with stunting, malaria parasitaemia or recent illness. The associations of underlying causes of anaemia on which the program acted (water and sanitation) and basic causes such as socioeconomic status were negligible (data not shown).

**Figure 3:**
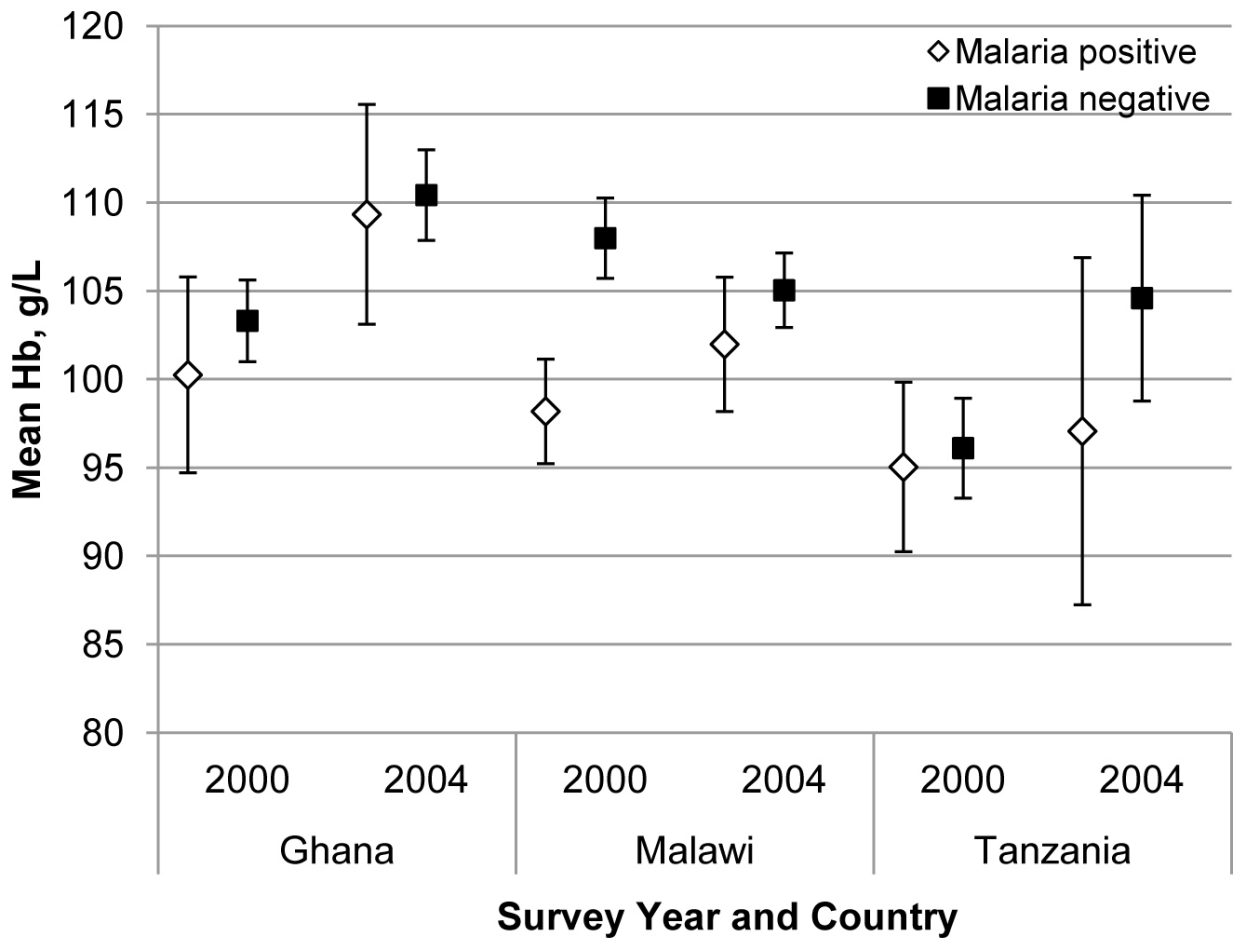
Comparison of mean haemoglobin with 95% CI among children with and without malaria parasitaemia by year and country.

Examination of two-way interaction terms for survey year with stunting, malaria and recent illness in each country dataset revealed evidence for variation between years for the association with haemoglobin of malaria and recent illness but not stunting. Figure 3 presents a comparison of the marginal means across countries and years for children with and without malaria parasitaemia. Malawian children with malaria parasitaemia had lower mean haemoglobin levels (difference -9.81 g/L; 95% CI −13.02, −6.59; p<0.001) compared to those without malaria in 2000, but this large deficit was no longer evident in 2004 (difference -3.06 g/L; 95% CI −6.69, 0.57; p = 0.098), due in part to an observed tendency for a higher mean haemoglobin level among children with malaria in 2004. There was no association of malaria status with mean haemoglobin in Ghana or Tanzania, in part due to wide confidence intervals for these estimates.

**Figure 4:**
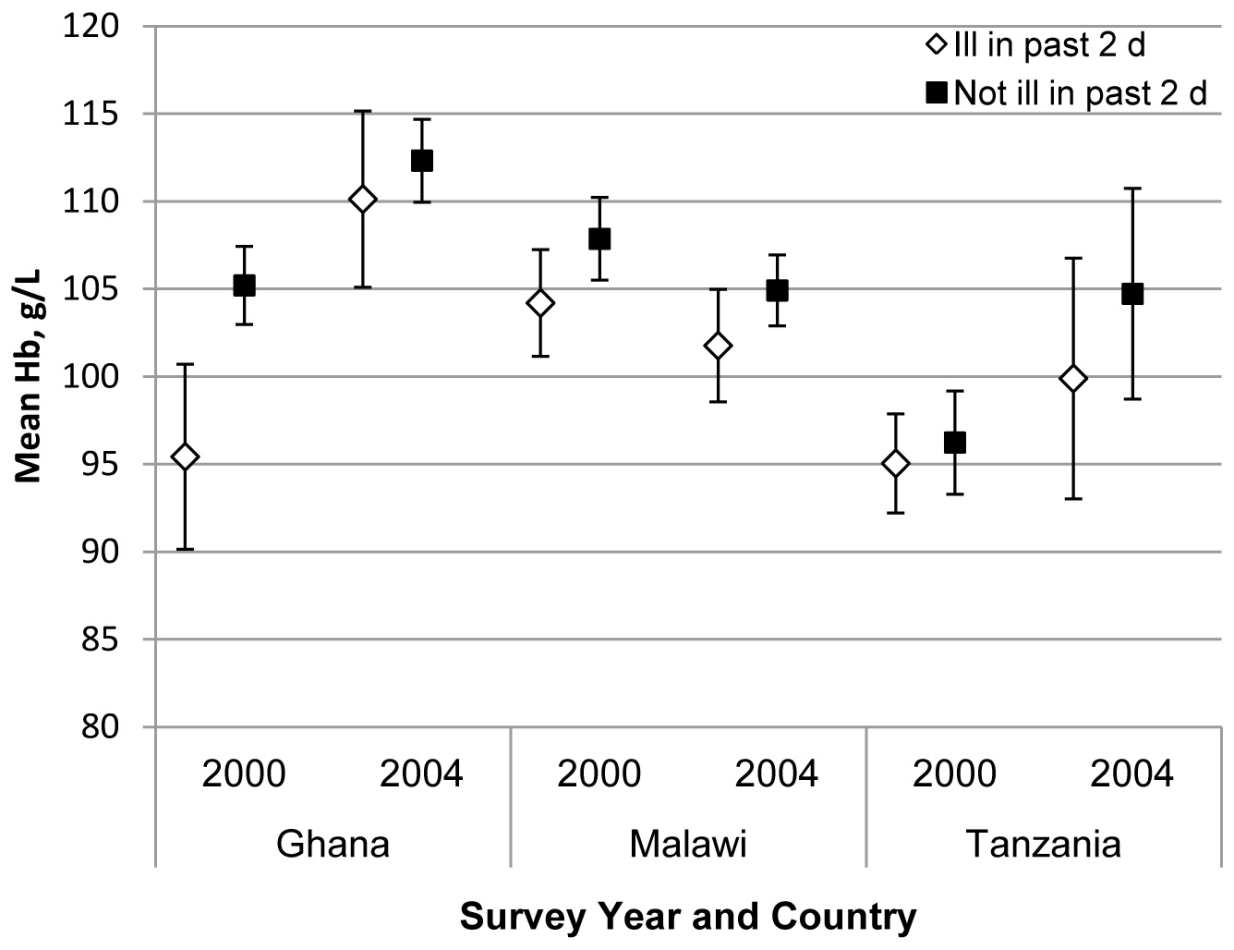
Comparison of mean haemoglobin with 95% CI among children with and without illness in the previous two days by year and country.

Evidence of variation between 2000 and 2004 in the association between recent illness and mean haemoglobin was found only in Ghana, as shown in Figure 4. In this country context, children who were reported to be ill in the past two days had a markedly lower mean haemoglobin level in 2000 compared to those who had not been ill (difference -9.78 g/L; 95% CI −15.20, −4.36; p<0.001). However, in 2004, the mean haemoglobin level of children who were ill in the past two days was much higher compared to those who were ill in 2000 (difference +14.70 g/L, 95% CI 7.52, 21.89; p<0.001) and no longer different from children in 2004 who were not ill (difference 2.19 g/L; 95% CI −2.80, 7.19; p = 0.389).

## Discussion

In this study, improvement in anaemia levels appeared to be primarily attributable to decreases in the prevalence of risk factors and not to reduction in the relative risks. Nevertheless, some evidence was observed for a buffering effect of improvements in health and nutrition occurring among young children and their families between 2000 and 2004, in terms of reducing the negative impact of risk factors for anaemia. Specifically, the children most vulnerable to anaemia – those with malaria – were less anaemic post-intervention in all three countries, suggesting they were more robust and less prone to the risk of complications or death associated with malaria. In addition, children who were ill in the past two days were less anaemic post-intervention, particularly in Ghana, and appeared more resilient to the negative effects that illness can have on haemoglobin levels.

Stunting was associated with a 3 g/L lower haemoglobin level, with no evidence for variation in this association between 2000 and 2004. Improvements in child health and nutrition, household food diversity, maternal knowledge of appropriate child care and feeding practices, as well as improvements in health care services and healthy environments were expected to contribute to enhanced child capacity to respond. However, the vulnerability to anaemia associated with stunting remained unchanged. No studies that we are aware of have looked at the change over time in the relationship between haemoglobin and stunting. However, a recent study modeling the risk of anaemia in preschool children in Ghana, Mali and Burkina Faso estimated that almost 40% of anaemia cases in 2011 would have been averted by improving the nutritional status of children [15]. The majority of cross-sectional studies from similar areas and age groups have shown lower mean haemoglobin levels and an increased risk of anaemia associated with stunting [16]–[20].

Malaria is a major contributor to anaemia, especially moderate and severe anaemia, in young children in African countries [15], [21]. Recent studies from West Africa [15] and Kenya [22], [23] estimate the proportion of anaemia attributable to malaria as approximately 15%. Malaria infection has a direct effect on a child's haematological status and the full consequences of malaria may be modified by several factors, including the child's nutritional status (especially iron status) before, during and after any clinical episode, the promptness and effectiveness of the treatment and the time for recovery prior to the next episode [24]. The relationship observed between haemoglobin and malaria in the Malawi samples was consistent with our hypothesis. A haemoglobin deficit of nearly 10 g/L associated with malaria infection among children in Malawi in 2000 was no longer evident in 2004. The reduction in magnitude of the lower haemoglobin associated with malaria between 2000 and 2004 may be due to the reduction in exposure to malaria during this time period, the buffering effect of other interventions, or a combination of these.

Improvements in health and nutrition occurring among children and their families in Malawi program areas between 2000 and 2004 may have provided a buffering effect, contributing to a decrease in children's vulnerability to the anaemia-producing effects of malaria, despite ongoing exposure to the disease. Improved child nutrition, as evidenced by a lower prevalence of underweight in 2004, may have contributed to reduced malaria-associated morbidity. Since a large proportion (almost 60%) of preschool children in Malawi have iron deficiency anaemia (National Micronutrient Survey in Malawi, 2001) and three-quarters of the children surveyed were benefiting from iron supplementation in 2004, iron supplementation may have contributed to improved iron status in these children, resulting in a decrease over time in the haemoglobin deficit associated with malaria, especially chronic malarial infection. Data available in 2004 showed no association between reported access to weekly iron supplementation for a child under five years of age in the household and the child's anaemia or malaria status. The inflammatory response during acute malaria episodes as well as during chronic low-density asymptomatic parasitaemia blocks iron absorption and utilization, limiting a child's response to supplemental iron [25]. A large trial in Zanzibar found an increased risk of adverse effects associated with iron and folic acid supplementation in children 1-35 mo [26], but a substudy of the same trial reported that children who were iron deficient or anaemic at baseline experienced significant benefits from iron and folic acid supplementation relative to their placebo-supplemented peers [27].

Improved access to treatment for malaria through program-supported village health revolving funds and community health education on the importance of prompt and effective care-seeking behaviours may have contributed to a decrease in severity of anaemia associated with malaria, as found in other studies in Africa [28], [29]. Malawian children living in rural locations are less likely to receive prompt, appropriate treatment for fever illness [30]. MICAH Malawi 2004 survey results showed a higher proportion of children in MICAH program areas compared to non-program areas who had malaria in the two weeks preceding the survey and received malaria treatment (66% vs. 45%, p<0.05) [31].

The program may have also successfully reduced co-infection rates through deworming and water, sanitation and hygiene promotion activities. Concurrent helminth infections make the immune response to malaria more inflammatory in young children [32]. A reduction in levels of co-infection may lessen the severity of the inflammatory response to malaria in children and lower negative impact on haemoglobin levels. Co-infections of hookworm and either schisto or trichuris also have been shown to have a synergistic effect, associated with higher levels of anemia than would be expected if the effects of these species had only independent effects on anemia [33]. Although data were not available on individual child hookworm infection status or time since most recent deworming medicine received, the program supported mass deworming of all children 2–5 y every six months.

Recent illness was associated with a lower haemoglobin of nearly 3 g/L overall, with evidence in Ghana for a buffering of this relationship between the two time points. Although children living in Ghana program areas experienced a similar prevalence of common illnesses in 2004, they may have responded differently to that exposure due to improved immunity and nutritional status and improved sick child caring practices, including increased health service utilization. Although the anaemia of inflammation is commonly short-term and of mild severity [34], it can be associated with more severe and chronic anaemia in contexts of widespread iron deficiency, frequent illness and high levels of stunting. In the pooled sample in our study, the magnitude of association (3 g/L) is comparable to that observed in other cross-sectional studies. In a study in Kenya among children 0–36 mo, a model that included malaria parasitaemia (as our model did) showed a lower mean haemoglobin of 3.1 g/L for children with diarrhoea in the last two weeks, 6.4 g/L for fever in the last two weeks and 7.9 g/L for current fever [17]. Two studies from other regions also reported a lower mean haemoglobin of 2.1 g/L among children with diarrhoea in the previous two weeks [35], [36].

Although the proportion of children recently ill was lower in 2004 in Malawi and Tanzania, suggesting reduced exposure, reduced duration of illness and/or improved disease control in these areas as compared to 2000, little buffering of MICAH program interventions was observed in terms of decreasing the risk of anaemia associated with recent illness in these two countries. Given the high levels of stunting observed in these children, it is likely that they were suffering from multiple nutritional deficiencies. Stunted children respond in different ways than non-stunted children to interventions; the duration of benefit from combined vitamin A supplementation and deworming was less in stunted children [37]. In the Philippines, improved retinol levels persisted for a much shorter time among stunted compared to non-stunted children [38]. These results suggest that the level of buffering that health and nutrition interventions have on child vulnerability may vary based on the factors underlying child stunting.

Another possible reason for observing a change in this risk relationship only in Ghana is that the inflammation associated with infections in these children is unavoidable, no matter what is the child's capacity to respond. In this case, program interventions can only hope to reduce exposure to pathogens and improve case management to ensure rapid recovery of haemoglobin levels, rather than expect to avoid the inflammation-associated decline in haemoglobin during illness. The inflammatory response that is triggered following an external or internal inflammatory stimulus is a normal function of the body's innate immune system and plasma concentrations of several nutrients, including iron, fall rapidly, irrespective of nutritional status [34]. This decrease in nutrient availability may have more serious functional consequences for children with prolonged infection and those who were already malnourished at the outset, including a reduced ability to deal with the infection. However, there is general consensus that the mild anaemia associated with an inflammatory response may be beneficial or protective, particularly for children living in areas with high exposure to infection [27], [34], [39]. Further elucidation of these mechanisms and their relevance in contexts with high levels of child malnutrition and frequent illness is needed.

Children in the youngest age group continued to be at higher risk of low Hb, regardless of country or survey year, and the magnitude of association with haemoglobin for age 48–59 mo (vs. 24–35 mo) was larger than for the three immediate causes (stunting, malaria, recent illness). Since program interventions commenced in most areas in 1998–99, children 24–59 mo assessed in 2004 were expected to have been exposed to the integrated health and nutrition program interventions throughout their early years, the period of greatest vulnerability and the period during which the largest response to intervention was expected [40]. The program's focus on enhanced maternal nutrition was expected to improve the child's health and nutritional status from infancy. Despite these efforts, no change over time in the age-anaemia risk relationship was observed. Longitudinal studies in African contexts have consistently shown an age-related evolution of anaemia, with children 6–30 mo at highest risk and a gradual decrease in risk thereafter [41], [42]. Cross-sectional studies also have highlighted the decrease in risk of anaemia among children over two years of age [15], [16], [20], [21], [43]–[45] and the tendency for higher prevalence of severe anaemia in children under two years of age [46].

One possible reason for the stable relationship between age and Hb levels observed in our study is that the key period of vulnerability (0–23 mo) for the development of iron and other nutrient deficiencies was not adequately addressed by program interventions designed to improve infant and young child feeding practices. Although program evaluation results showed increased exclusive breastfeeding rates in all three country contexts during this period of time, little change was found in the types of complementary foods given to children [31], [47], [48]. Therefore, it is unlikely that the changes observed during this period of time were of sufficient magnitude to effect a change in the age-anaemia risk relationship. Furthermore, recent evidence suggests that even highly effective complementary feeding interventions may not be sufficient to prevent all stunting, based on observations in Malawi that 40% of the cumulative deficit in stature occurred before six months of age [49]. Improving the micronutrient status of women during pregnancy through multiple micronutrient supplementation can also contribute to meaningful improvements in the growth and development of children by two years of age [50].

### Limitations of our study

Although the package of nutrition and health interventions delivered in the study areas by the MICAH program is very similar to the recommended list of effective interventions to reduce child undernutrition [51], and was also aligned with the context-specific causes of anaemia, one major limitation of this study is that the coverage achieved for each intervention varied widely and we were unable to assess the extent to which each child in the study was reached with the interventions delivered. Based on the coverage data summarized as part of the adequacy evaluation of the program [Table 5 in 10], coverage levels of around 70% or higher were achieved in all three countries for most anaemia-related interventions measured, with the exception of child insecticide-treated bednet use in Ghana and Tanzania, and child iron supplementation and access to potable water sources in Tanzania. However, this did not include measures of coverage for dietary diversification strategies, infant and young child feeding education strategies, growth monitoring, deworming of children under five years of age and other specific disease control strategies (e.g. malaria treatment for children under five years of age with fever). For many of these strategies, the review of process indicators showed relatively low to moderate coverage of targeted groups.

While the variables in our models may have been measured with a minimum of error, they still reflect imprecisely the constructs they are intended to represent. Stunting is an indicator of multiple constraints to health but does not reflect precisely the multiple dimensions of nutritional status, and malaria and recent illness are only two aspects of morbidity relevant to the study of child haemoglobin in these contexts. The cross-sectional nature of the data did not allow us to ascertain causal relationships and therefore we cannot make any statement about what direction of effect occurred in the associations observed. Conducting *post hoc* analysis precluded data collection for variables of particular importance to the research questions being asked, such as measures of child iron status and dietary intake, vitamin A status, inflammation, helminth infection, malaria parasite density, HIV status and genetic diseases known to be associated with anaemia (e.g. sickle cell anaemia). The absence of a control group also precludes us from ascribing any observed changes to the MICAH program per se.

## Conclusion

In areas where multiple interventions were implemented to decrease the prevalence of anaemia, some buffering of malaria and recent illness occurred, consistent with the expected positive impact of decreased child vulnerability to the anaemia-producing effects of acute illness. Overall impact on anaemia rates was likely due to a combination of reduced prevalence of morbidity and reduced vulnerability. Stunting, however, remained an unbuffered risk factor for anaemia. Effectively reducing the causes of chronic undernutrition is required in order to further reduce child vulnerability and ensure maximum impact of anaemia control programs. Additional research on the utility of the child vulnerability concept in the field of public health nutrition, and particularly in the analysis of anaemia program effectiveness, is recommended.

## Supporting Information

Table S1 **Summary of MICAH interventions by country.**
(DOCX)
