# Supplementary material for: Can an Integrated Approach Reduce Child Vulnerability to Anaemia? Evidence from Three African Countries
Source: PLoS One. 2014 Mar 5;9(3):e90108. doi: 10.1371/journal.pone.0090108 (PMC3943899; doi:10.1371/journal.pone.0090108)
Supplement: Table S1 — Summary of MICAH interventions by country. (DOCX) [file pone.0090108.s001.docx]

**Table S1:** Summary of MICAH interventions by country^a,b^

| **Objective** | **Strategy** | **Target Group/Activity** | **Ghana** | **Malawi** | **Tanzania** |
| --- | --- | --- | --- | --- | --- |
| Increase intake and bioavailability of micronutrients (iron, iodine and vitamin A) | Vitamin A supplementation | Preschool children | P,D,T,A,M |  | D,T,M |
|  |  | School age children |  |  |  |
|  |  | Postpartum women | P,D,T,A,M |  | D,T,M |
|  | Iron supplementation | Preschool children | P,D,T,A,M | P,D,T,A,M | P,D,T,M |
|  |  | School age children |  |  |  |
|  |  | Women of childbearing age |  | P,D,T,A,M |  |
|  |  | Pregnant women |  |  | P,D,T,M |
|  | Fortification | Iodized salt promotion | P,D,T,A,M | T,A,M | T,M, A |
|  |  | Small scale flour fortification |  | P,D,T,A, M | P,D,T,A,M |
|  | Dietary Diversification | Small animal rearing | P,D,T,M | P,D,T,M | P,D,T,M |
|  |  | Vegetable gardens |  |  |  |
|  |  | Fruit tree cultivation |  |  |  |
|  | Infant & Young Child Feeding | Promotion of optimal breastfeeding | T,M, A | T, M, A | T, M, A |
| Reduce prevalence of diseases that affect micronutrient status (diarrheal, parasitic and vaccine-preventable) | Water and Sanitation | Provision of clean water | P,D,T,M | P,D,T,M | P,D,T,M |
|  |  | Latrine construction |  |  |  |
|  |  | Garbage disposal construction | T,M | T,M | T,M |
|  | Malaria Control | ITNs distribution | P,D,T,M | P,D,T,M | P,D,T,M |
|  |  | Chemoprophylaxis to pregnant women | M | P,D,T,M | P,D,T,M |
|  |  | Malaria treatment to preschool children | T |  |  |
|  | Treatment of Worms and Parasites | Deworming of preschool children | P,D,T,M | P,D,T,A,M | P,D,T,M |
|  |  | Deworming of school age children |  |  |  |
|  |  | Schistosomiasis treatment |  |  |  |
|  | Immunization | Support EPI campaigns | P,D,T,M | P,D,T,M | D,T,M |
|  | Diarrhea Treatment | Provision of ORT | T | P,D,T | T |
|  | HIV/AIDS prevention | IEC re: HIV and AIDS | T | T | T |
| Build local capacity for delivery systems to improve micronutrient status | Education | Health and nutrition IEC to communities | D,T,M | D,T,M | D,T,M |
|  |  | Staff and partner training | T | T | T |
|  | Advocacy | Influence national policies on nutrition issues | A | A | A |
|  |  | Use of media to communicate nutrition and health messages | T,A | T,A | T,A |

^a^ Adapted from Berti et al., 2010

^b^ Blank cells indicate that MICAH did not work on that target group/activity in that country.

**P**: procurement; **D**: delivery/distribution (includes both transport to distribution sites such as health centres and direct delivery to beneficiaries); **T**: training (includes both community education and training of implementing staff/volunteers); **A**: advocacy; **M**: monitoring

ITN=insecticide treated bednet; EPI=expanded program on immunisation; ORT=oral rehydration therapy; IEC=Information, Education and Communication
